# Salivary and serum levels of NT-proBNP, troponin, and high-sensitivity C-reactive protein in patients with heart failure

**DOI:** 10.1371/journal.pone.0357206

**Published:** 2026-09-18

**Authors:** Ruy Felipe Melo Viegas, Sheila Cavalca Cortelli, Davi Romeiro Aquino, Fernando Oliveira Costa, Bruna Hentges, Vitórya Carvalho Pádua de Magalhães, Rodrigo Augusto Foganholi Silva, Gilson Fernandes Ruivo, Jose Roberto Cortelli

**Affiliations:** 1 Department of Medicine, University of Taubaté, Taubaté, São Paulo, Brazil; 2 Department of Dentistry, University of Taubaté, Taubaté, São Paulo, Brazil; 3 Department of Clinical Sciences, Pathology, and Surgery, Federal University of Minas Gerais, Belo Horizonte, Minas Gerais, Brazil; 4 School of Nursing and Public Health, Federal University of Rio Grande do Sul, Porto Alegre, Rio Grande do Sul, Brazil; 5 CEEpiRG - Center for Epigenetic Study and Genic Regulation, Program in Environmental and Experimental Pathology, Paulista University, São Paulo, São Paulo, Brazil; Virginia Commonwealth University, UNITED STATES OF AMERICA

## Abstract

**Background:**

Saliva has been proposed as a non-invasive biological matrix for cardiovascular biomarker assessment. However, evidence regarding its agreement with blood-based measurements in patients with heart failure remains inconsistent.

**Objective:**

To assess the presence of NT-proBNP, troponin, and high-sensitivity C-reactive protein in saliva and their correlation with serum concentrations in patients with heart failure over a six-month follow-up period.

**Methods:**

Participants were prospectively evaluated, and paired saliva and blood samples were collected at baseline, 3 and 6 months. The population included 52 individuals (30 males and 22 females) with HFmrEF (LVEF 41–49%) or HFrEF (LVEF ≤40%), aged 49–80 years (mean age of 61.1 ± 6.79 years). Biomarker concentrations were measured in both matrices. Agreement between salivary and serum measurements was assessed using Bland–Altman analysis. Additional analyses explored whether age influenced the relationship between salivary and serum biomarker levels.

**Results:**

All three biomarkers were consistently detectable in whole saliva. However, Bland–Altman analyses showed that salivary NT-proBNP and troponin did not demonstrate clinically acceptable agreement with serum concentrations at any evaluated time point, indicating limited interchangeability between matrices. In contrast, hs-CRP exhibited relatively better agreement between saliva and serum, with mean bias close to zero and moderate limits of agreement, although substantial inter-individual variability persisted. Age and sex did not meaningfully influence the relationship between salivary and serum biomarker concentrations.

**Conclusions:**

Although NT-proBNP, troponin, and hs-CRP are detectable in saliva, salivary measurements of NT-proBNP and troponin showed insufficient agreement with serum levels to support their use as substitutes for blood-based assessment in heart failure. hs-CRP demonstrated comparatively better performance but remains limited by variability. Under the conditions evaluated, serum remains the reference standard for cardiovascular biomarker assessment in heart failure, and saliva should be regarded as a matrix warranting further investigation.

## Introduction

Heart failure (HF) represents an escalating global public health problem, associated with substantial morbidity and mortality, as well as a considerable economic impact on healthcare systems worldwide [1]. Data from the Global Burden of Disease (GBD) study has been instrumental in characterizing the epidemiological profile of HF, providing comprehensive estimates of prevalence, mortality, and disability-adjusted life years (DALYs) across diverse regions and populations [2].

HF is estimated to affect nearly 64 million individuals worldwide, with a substantial proportion of this burden concentrated in low- and middle-income countries [3].

Circulating biomarkers have become valuable tools for improving the diagnosis, risk stratification, and clinical management of patients with HF. Among these, natriuretic peptides (NPs) were the earliest molecules identified as effective tools for HF management and continue to play a central role in contemporary HF guidelines [4,5]. These peptides are circulating cardiac biomarkers of myocardial stretch and biomechanical stress and comprise different molecular forms, including B-type natriuretic peptide (BNP) and N-terminal pro–B-type natriuretic peptide (NT-proBNP). In addition, cardiac troponin is an integral biomarker in the evaluation and management of patients with acute coronary syndrome. Troponin is also established as a valuable prognostic marker in patients with acute or chronic HF [6].

Elevated inflammatory activity is frequently observed in patients with HF and has been consistently associated with an increased risk of adverse outcomes, including mortality. Among the inflammatory biomarkers, high-sensitivity C-reactive protein (hs-CRP) reflects the degree of systemic inflammation and can be easily measured in clinical practice.

According to Kumari et al., human saliva reflects the body’s health and overall well-being. More than 20% of proteins present in blood are also detectable in saliva, characterizing saliva as a non-invasive alternative for the identification of biomarkers correlated with disease status. Compared with blood, saliva offers several advantages as a diagnostic fluid because it enables non-invasive, simple, and safe sample collection. Its biochemical composition reflects the individual’s physiological and pathological states, supporting its potential use as a diagnostic medium for systemic diseases [7].

We hypothesize that, in patients with HF, NT-proBNP, troponin, and hs-CRP are present in saliva and correlate with their respective serum concentrations, supporting saliva as a potential non-invasive alternative for cardiovascular assessment. Accordingly, this prospective longitudinal study aimed to evaluate the presence of these biomarkers in saliva and to assess the correlation between their salivary and serum concentrations over a six-month follow-up.

## Materials and methods

Participants included in the present study were recruited from the Municipal University Hospital of Taubaté (HMUT), Taubaté, Brazil, within an ongoing clinical research program involving patients with heart failure conducted at this tertiary care center. Initially, an observational study was approved by the local Research Ethics Committee (CAAE: 63946522.7.0000.5501; approval number 5.708.195) and initiated in 2023 to identify and characterize eligible patients. Subsequently, participants were enrolled in an interventional clinical study approved under a second ethical protocol (CAAE: 76621623.3.0000.5501; approval number 6.651.994). The present manuscript reports a secondary analysis derived from this interventional cohort. The study was registered at ClinicalTrials.gov (NCT07036289) and was conducted in accordance with the principles of the Declaration of Helsinki. All participants received detailed oral explanations about the study procedures, and written informed consent was obtained prior to their inclusion in the study.

### Study design and patient recruitment

This was a prospective longitudinal observational study conducted in patients with HF. Among 94 consecutive individuals assessed for eligibility, 52 patients were enrolled between 10/June/2025 and 30/June/2025. All participants had HF with mildly/reduced ejection fraction were under guideline-directed medical therapy for heart failure with reduced or mildly reduced ejection fraction, according to contemporary international recommendations. Optimized pharmacological treatment included a beta-blocker, inhibition of the renin–angiotensin system (angiotensin-converting enzyme inhibitor, angiotensin receptor blocker, or angiotensin receptor–neprilysin inhibitor), a mineralocorticoid receptor antagonist, and a sodium–glucose cotransporter-2 inhibitor when clinically indicated and tolerated. Diuretics were prescribed when necessary for the management of congestion. Pharmacological treatment was supervised by the attending cardiologist in accordance with current heart failure guidelines [5]. Health status related to heart failure was assessed using the Kansas City Cardiomyopathy Questionnaire (KCCQ), a validated disease-specific instrument that evaluates physical limitation, symptom burden, quality of life, and social limitation in patients with heart failure. Scores range from 0 to 100, with higher scores indicating better health status. Clinical interpretation thresholds classify scores as severe (<25), moderate-to-severe (25–49), moderate (50–74), and good to excellent health status (≥75) [8,9]. No participant changed KCCQ category during the follow-up period. All participants

The study population was followed over a 6-month period, with evaluations performed at baseline, 3 months, and 6 months. At each study visit, clinical and cardiological data were collected, and paired blood and saliva samples were obtained for biomarker analysis.

### Sample size considerations

The sample size was originally estimated for the primary objective of the clinical study from which this dataset was derived, which evaluated the effects of periodontal treatment on clinical parameters and systemic biomarkers in patients with heart failure. The calculation was based on expected differences in BNP and NT-proBNP levels and followed the formula MSD = t(5%) √((2 × SD)/N), where MSD represents the minimum significant difference, t(5%) corresponds to the tabulated value for a 5% significance level, SD represents the observed standard deviation, and N the required sample size. A minimum detectable difference of 10% was assumed.

Based on these parameters and including a 10% safety margin, the estimated sample size was approximately 25 participants per group (treatment and control). The present study represents a secondary analysis of this cohort and included all available participants with heart failure (n = 52), regardless of their original group allocation, since the objective was to evaluate the agreement between salivary and serum biomarker measurements.

### Inclusion and exclusion criteria

All participants had heart failure with mildly reduced or reduced ejection fraction (HFmrEF/HFrEF) [4–7]. Individuals with HFmrEF (left ventricular ejection fraction [LVEF] 41–49%) or HFrEF (LVEF ≤40%) were consecutively selected regardless of sex, and those who voluntarily agreed to participate were included according to the following eligibility criteria: (a) age ≥ 45 years; (b) feasibility and availability to undergo clinical examination; and (c) presence of stage II or III periodontitis according to the 2018 classification of periodontal diseases [10].

Exclusion criteria were: (a) pregnancy or lactation; (b) stage D heart failure requiring treatment with vasoactive drugs or circulatory assist devices; (c) heart failure with preserved ejection fraction (HFpEF; LVEF ≥50%); (d) reduced salivary flow or inability to provide a saliva sample; and (e) current use of tobacco products, including conventional cigarettes and electronic cigarettes.

### Blood sample collection

Blood samples were collected according to hospital protocols and nursing best practices in an outpatient setting. Following international recommendations from the Clinical & Laboratory Standards Institute, after antisepsis and tourniquet application to the participant’s arm, peripheral blood samples were drawn into pre-labeled 10 mL vacuum tubes for laboratory analysis.

After venous blood collection, samples were allowed to clot at room temperature and were subsequently centrifuged to obtain serum. Serum samples were aliquoted and stored at −80 °C until analysis, avoiding repeated freeze–thaw cycles.

### Serum biomarker analysis

Serum NT-proBNP levels were measured using an electrochemiluminescence immunoassay (ECLIA) (ProBNP, PNBR). Serum high-sensitivity cardiac troponin T (hs-cTnT) concentrations were also determined by electrochemiluminescence immunoassay (Troponin, TRP1/TR). Serum hs-CRP levels were quantified using an immunoturbidimetric assay (PCRU). All analyses were performed according to standardized laboratory protocols and the manufacturers’ recommendations.

### Saliva sample collection

A standardized protocol for the collection of unstimulated whole saliva was applied, in accordance with previously described guidelines [11]. Saliva samples were collected in the morning, between 8:00 and 11:00 a.m., and participants were instructed not to eat or drink prior to the procedure. Before collecting, individuals rinsed their mouths with water. During sampling, participants remained seated with their heads slightly tilted (approximately 45°) in a quiet environment. A total of 5.0 mL of unstimulated whole saliva was collected in sterile Falcon tubes. Samples were centrifuged at 15,000 × g for 10 minutes at 4°C, and the supernatant was stored at −80°C. All samples were processed following the same standardized protocol and stored under controlled conditions to minimize pre-analytical variability.

### Salivary biomarker analysis

Salivary biomarkers were quantified using commercially available enzyme-linked immunosorbent assay (ELISA) kits supplied by ELK Biotechnology, Sugar Land, TX, USA following the manufacturer’s instructions. Specifically, the Human NT-ProBNP (N-Terminal Pro-Brain Natriuretic Peptide) [Cód. ELK1212; Intra-assay Precision (CV% < 8%), Inter-assay Precision (CV% < 10%) and detection range (39.07–2500 pg/mL)]; Human hs-cTnT(High Sensitivity Cardiac Troponin T) [Cód. ELK8843; Intra-assay Precision (CV% < 8%), Inter-assay Precision (CV% < 10%) and detection range (15.63–1.000 pg/mL)] and Human hs-CRP (high sensitivity C Reactive Protein) [Cód. ELK8534; Intra-assay Precision (CV% < 8%), Inter-assay Precision (CV% < 10%) and detection range (1.57–100 ng/mL)] ELISA Kits were used for the determination of salivary concentrations of NT-proBNP, high-sensitivity cardiac troponin T, and high-sensitivity C-reactive protein, respectively.

All assays were performed under standardized laboratory conditions, and absorbance readings were obtained using a microplate reader at the wavelengths specified by the manufacturer. Concentrations of salivary biomarkers were calculated from standard calibration curves generated for each analyte. All samples were analyzed in duplicate, and the mean value of the duplicate measurements was used for statistical analyses to improve analytical precision.

The test principle applied in this kit is Sandwich enzyme immunoassay. The microtiter plate provided in this kit has been pre-coated with an antibody specific to Human NT-ProBNP. Standards or samples are added to the appropriate microtiter plate wells then with a biotin-conjugated antibody specific to Human NT-ProBNP. Next, Avidin conjugated to Horseradish Peroxidase (HRP) is added to each microplate well and incubated. After TMB substrate solution is added, only those wells that contain Human NT-ProBNP, biotin-conjugated antibody and enzyme-conjugated Avidin will exhibit a change in color. The enzyme-substrate reaction is terminated by the addition of sulphuric acid solution, and the color change is measured spectrophotometrically at a wavelength of 450nm ± 10nm. The concentration of Human NT-ProBNP in the samples is then determined by comparing the OD of the samples to the standard curve.

### Statistical analysis

Descriptive analyses were conducted to summarize biomarker concentrations separately for each biomarker (NT-proBNP, troponin, and hs-CRP), biological matrix (serum and saliva), and follow-up time point (baseline, 3 months, and 6 months).

The normality of continuous variables was assessed using the Shapiro–Wilk test. Due to violations of normality assumptions, biomarker concentrations were log-transformed (log10) prior to analysis. All subsequent statistical analyses, including descriptive statistics, correlation analyses, regression models, and Bland–Altman agreement analyses, were performed using log10-transformed biomarker concentrations. Variables were summarized as mean and standard deviation when approximately normally distributed, or as median and interquartile range when skewness persisted after transformation.

The association between salivary and serum biomarker concentrations was evaluated using Spearman’s rank correlation coefficient. Ninety-five percent confidence intervals were estimated via bootstrap resampling (1,000 iterations) using the bias-corrected and accelerated (BCa) method.

Agreement between blood and saliva biomarker measurements was assessed using Bland–Altman analysis applied to log10-transformed values [12]. For each biomarker and sampling time, the difference between paired measurements (blood minus saliva) was plotted against their meaning. The mean difference was used to estimate systematic bias, and the 95% limits of agreement were calculated as the mean difference ± 1.96 standard deviations of the differences. Ninety-five percent confidence intervals for both the mean bias and the limits of agreement were estimated to quantify the precision of these parameters. Visual inspection of Bland–Altman plots was performed to assess the normality of the differences, the presence of proportional bias, and the homogeneity of variance across the measurement range.

To assess whether age modified the association between salivary and serum biomarker concentrations, linear regression models fitted including an interaction term between salivary biomarker concentration (log10-transformed) and age, modeled as a continuous variable centered at the mean. Models were estimated separately for each biomarker and sampling time. Effect modification was evaluated based on the interaction coefficient, its 95% confidence interval, and the corresponding p-value.

All analyses were conducted using a two-sided significance level of 5% and performed with the Statistical Package for the Social Sciences (SPSS), version 24.0. Analytical strategies were selected to ensure robust evaluation of the relationship between biological matrices, including non-parametric correlation methods with bootstrap confidence intervals and Bland–Altman analysis for agreement assessment.

## Results

**Fig 1:**
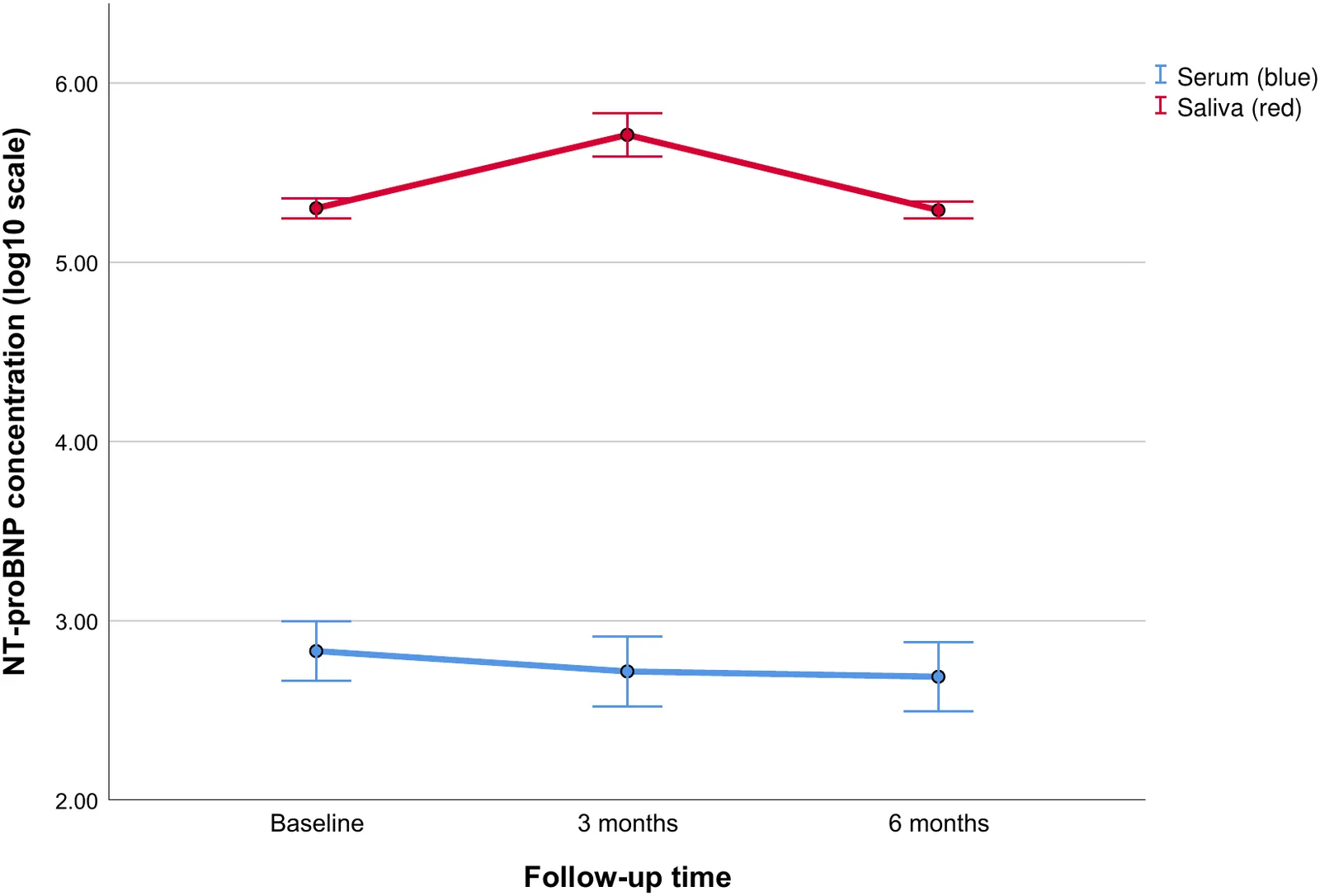
Longitudinal trends of serum and salivary NT-proBNP biomarker concentrations in patients with heart failure.

**Fig 2:**
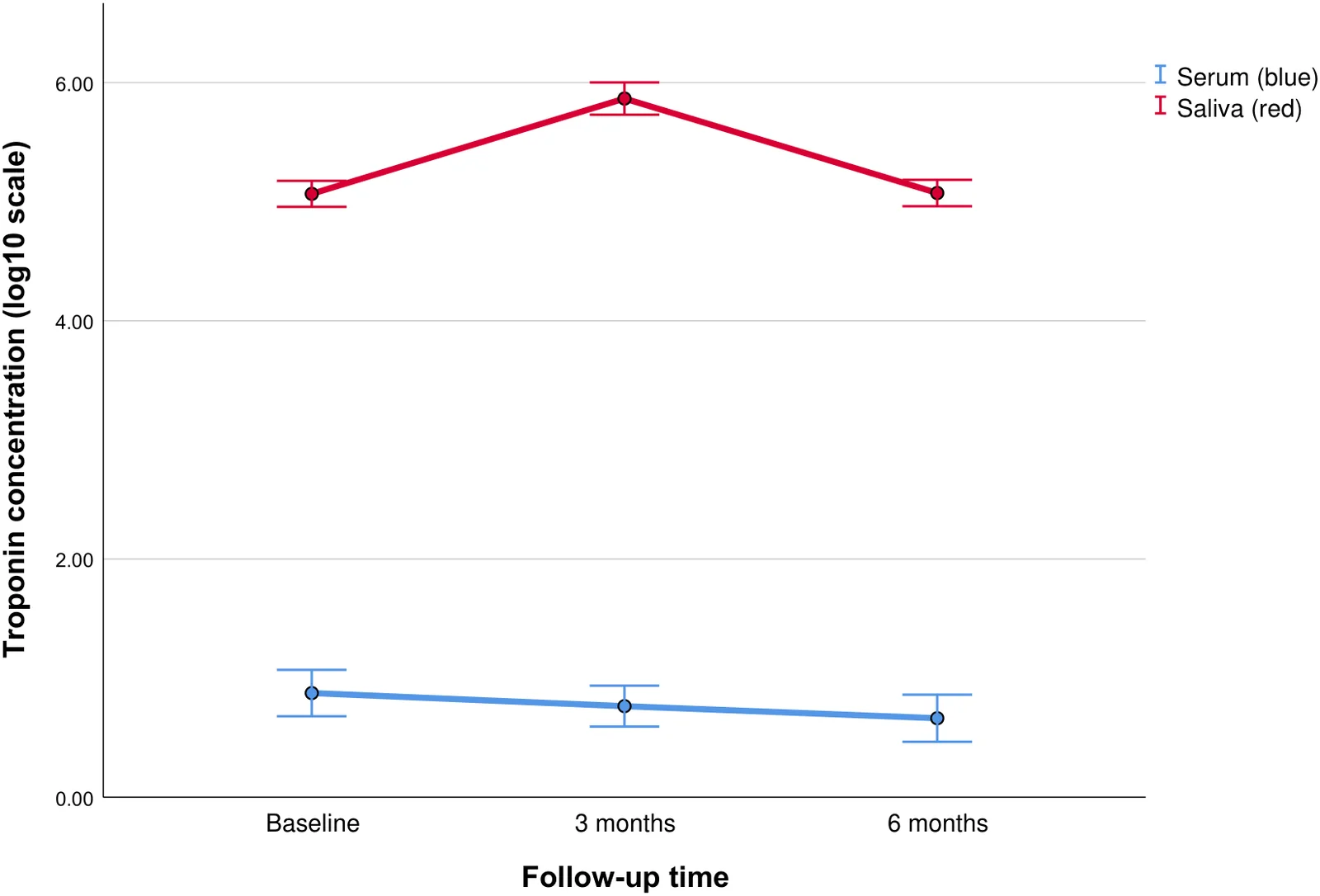
Longitudinal trends of serum and salivary Troponin biomarker concentrations in patients with heart failure.

**Fig 3:**
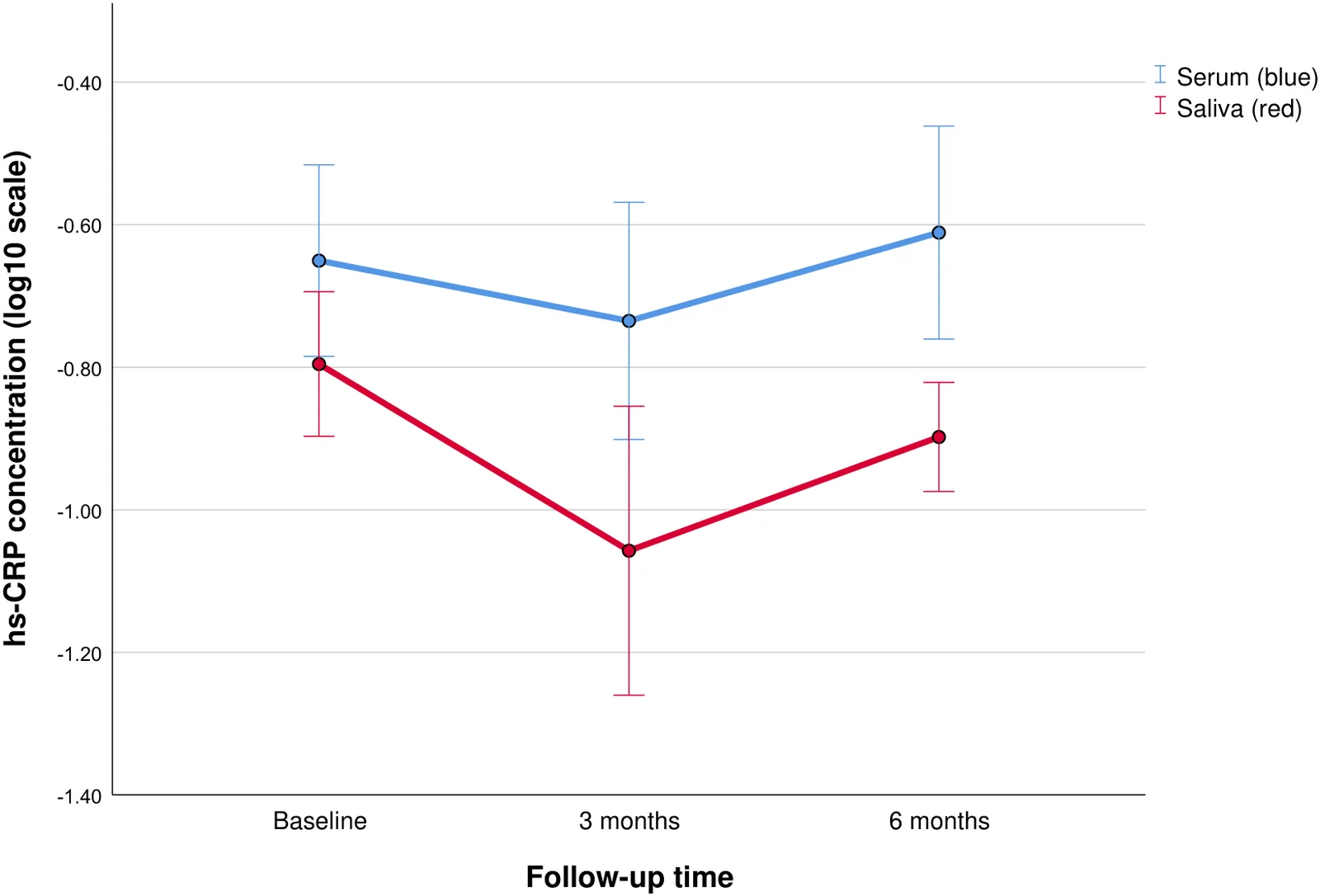
Longitudinal trends of serum and salivary hs-CRP biomarker concentrations in patients with heart failure.

The present study included a total of 52 participants aged 49–80 years. Of these, 30 were males (57.7%) and 22 were females (42.3%). The mean age of the participants was 61.1 ± 6.79 years; 61.93 ± 6.81 years (males) and 60.14 ± 6.78 years (females). At baseline, the study population presented a mean salivary flow of 0.32 + − 0.10mL/min. Additionally, the mean whole-mouth plaque and gingival index were 78.46% and 57.03%, respectively. Based on KCCQ clinical interpretation thresholds, 9 patients (17.3%) moderate-to-severe impairment (KCCQ 25–49), 7 (13.5%) moderate impairment (50–74), and 36 patients (69.2%) showed good to excellent health status (≥75). In addition, 37 (71.2%) had sinus rhythm, 13 (25.0%) had atrial fibrillation, and 2 (3.8%) had atrial fibrillation with pacemakers. Patients received guideline-directed medical therapy for heart failure with reduced or mildly reduced ejection fractions according to contemporary international recommendations. Descriptive statistics for log10-transformed serum and salivary NT-proBNP, cardiac troponin, and hs-CRP at baseline, 3 months, and 6 months are presented in Table 1. Longitudinal trends of serum and salivary concentrations of NT-proBNP, troponin, and hs-CRP across baseline, 3 months, and 6 months are presented in Figs 1-3.

**Table 1:** Descriptive statistics of log10-transformed serum and salivary NT-proBNP, cardiac troponin, and hs-CRP concentrations at baseline, 3 months, and 6 months in patients with heart failure.

Variables*	Baseline (n = 52)	3 months (n = 52)	6 months (n = 52)
**Blood data**			
NT-proBNP	2.83 (0.60)	2.74 (0.70)	2.59 (0.69)
Troponin	0.77 (0.50-1.08)	0.66 (0.51-0.99)	0.66 (0.50-1.00)
hs-CRP	−0.65 (0.47)	−0.70 (0.60)	−0.62 (0.52)
**Salivary data**			
NT-proBNP	5.30 (0.20)	5.71 (0.42)	5.29 (0.16)
Troponin	4.91 (4.86-5.01)	5.84 (5.46-6.26)	4.94 (4.87-5.13)
hs-CRP	−0.81 (0.37)	−1.06 (0.70)	−0.90 (0.26)

* Values are presented as mean (standard deviation) for approximately normally distributed variables and as median (interquartile range) for skewed variables. All concentrations are presented on a logarithmic scale (log10).

Serum and salivary biomarker concentrations showed distinct distributional patterns, justifying the use of logarithmic transformation and non-parametric correlation analyses. For NT-proBNP and troponin, salivary measurements were numerically higher than serum measurements on the log-transformed scale, whereas hs-CRP showed comparable or slightly higher serum values (Table 1).

The association between salivary and serum concentrations (Table 2) was assessed using Spearman’s rank correlation coefficient. Overall, correlations were weak and inconsistent across biomarkers and follow-up times. For NT-proBNP, weak positive correlations were observed at baseline and at 3 months; however, the corresponding 95% confidence intervals included the null value, indicating no statistically significant association. At 6 months, the correlation coefficient was close to zero.

**Table 2:** Spearman correlation analysis between salivary and serum concentrations of NT-proBNP, troponin, and hs-CRP at baseline, 3 months, and 6 months of follow-up.

Biomarkers	ρ (Spearman)	95% CIs	p-values	N
***NT-proBNP***				
**Baseline**	0.244	−0.053-0.489	0,082	52
**3 months**	0.137	−0.143-0.401	0,346	49
**6 months**	−0.022	−0.303-0.271	0,883	48
***Troponin***				
**Baseline**	−0.103	−0.375-0.193	0,468	52
**3 months**	0.084	−0.220-0.386	0,565	49
**6 months**	−0.232	−0.501-0.083	0,113	48
***hs-CRP***				
**Baseline**	0.111	−0.155-0.327	0,436	51
**3 months**	0.031	−0.228-0.277	0,832	49
**6 months**	−0.270	−0.532-0.045	0,064	48

Spearman’s rank correlation coefficients (ρ) are shown with 95% confidence intervals (95% CI) estimated by bootstrap resampling (1,000 iterations, bias-corrected and accelerated method).

For cardiac troponin, correlation coefficients were near zero at all time points, suggesting the absence of a monotonic relationship between salivary and serum concentrations. Similarly, hs-CRP showed weak correlations at baseline and at 3 months, with a tendency toward a weak negative correlation at 6 months; however, confidence intervals again encompassed the null value (Table 2).

### Bland–Altman analysis

Given that correlation assesses only the strength and direction of association between variables and does not reflect agreement between measurement methods, agreement between salivary and serum biomarker concentrations was further evaluated using the Bland–Altman method [12].

**Fig 4:**
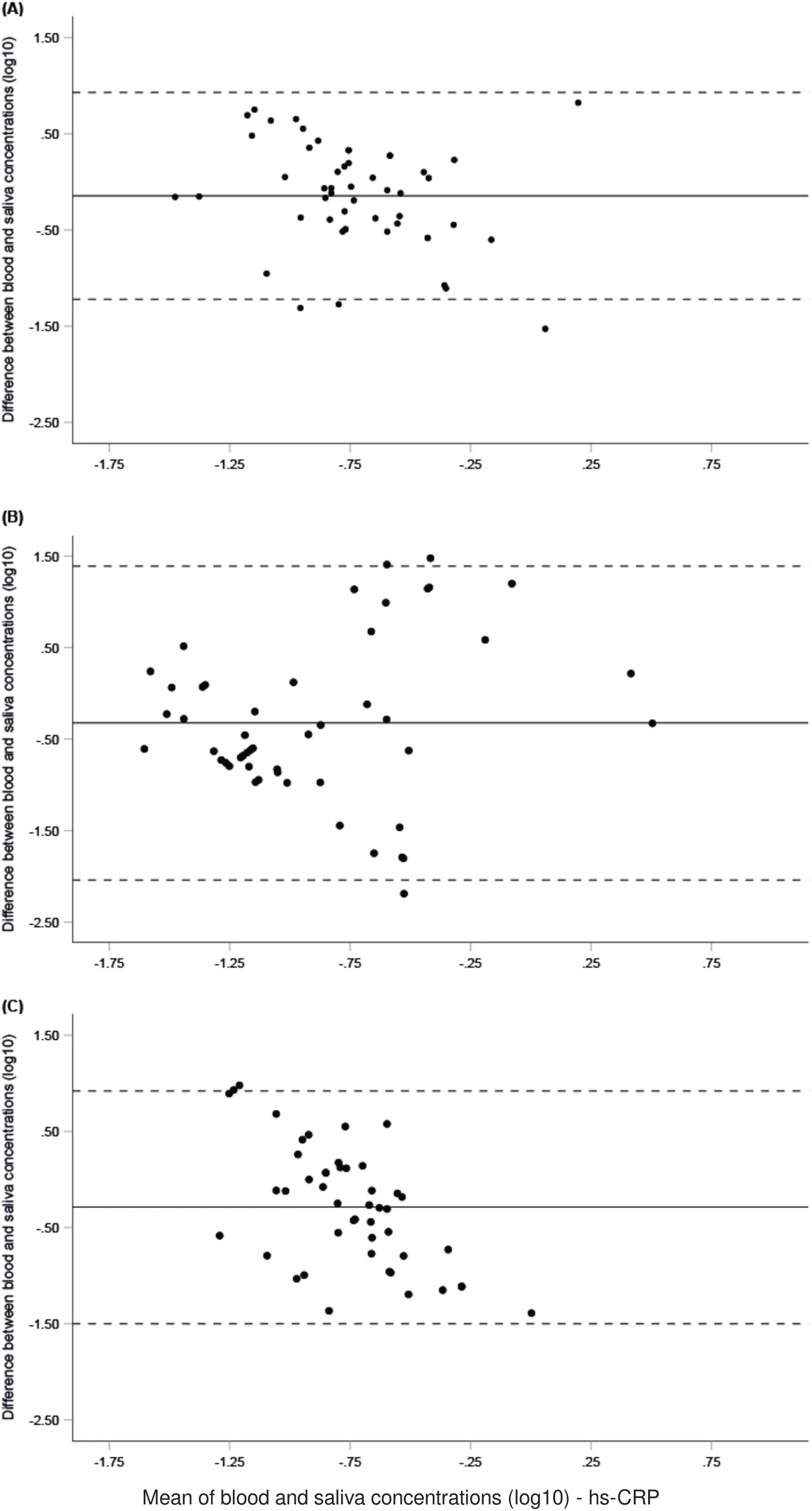
Bland–Altman plots showing agreement between serum and salivary NT-proBNP concentrations at baseline (A), 3 months (B), and 6 months (C).

**Fig 5:**
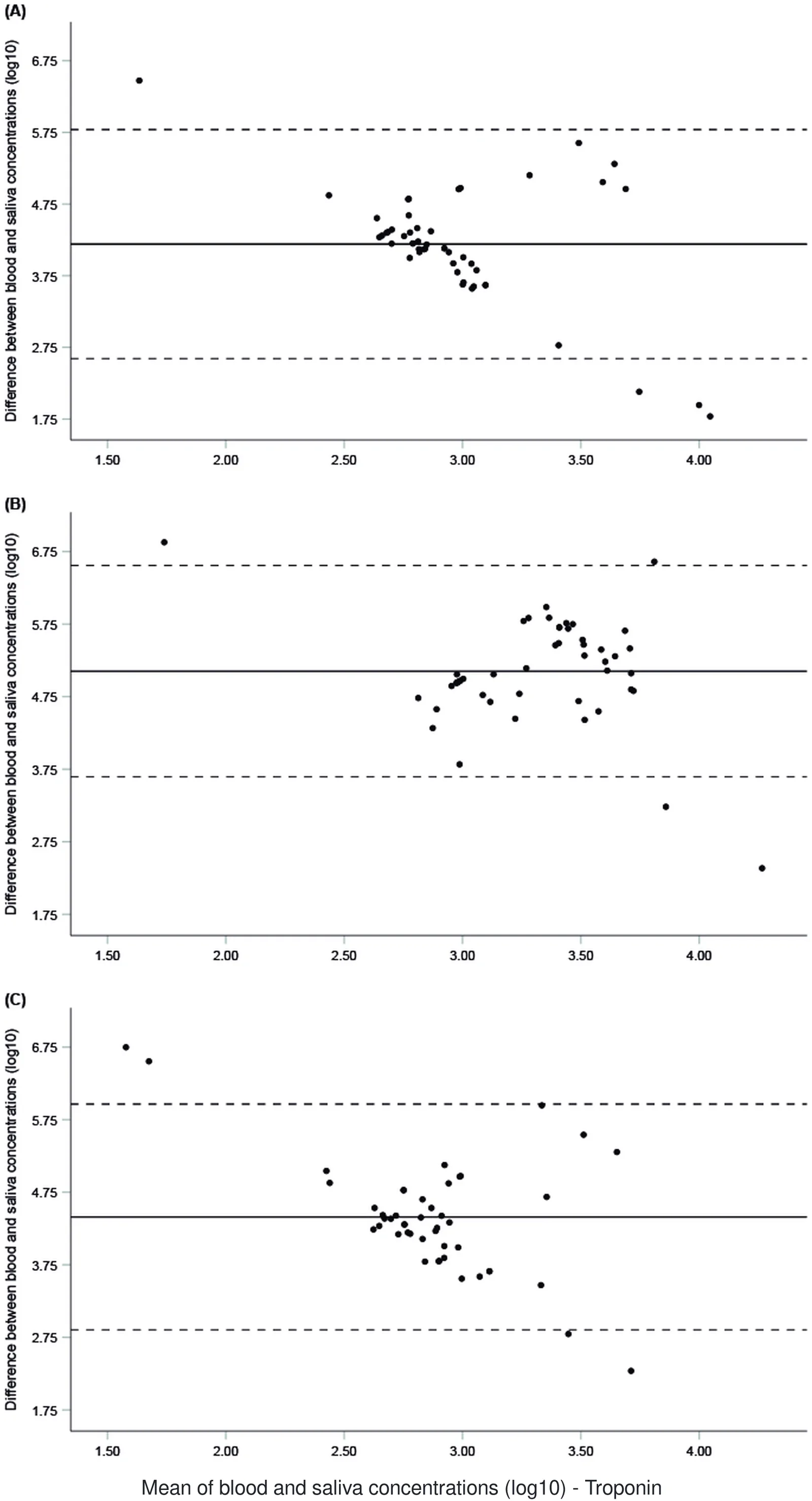
Bland–Altman plots showing agreement between serum and salivary cardiac troponin concentrations at baseline (A), 3 months (B), and 6 months (C).

**Fig 6:**
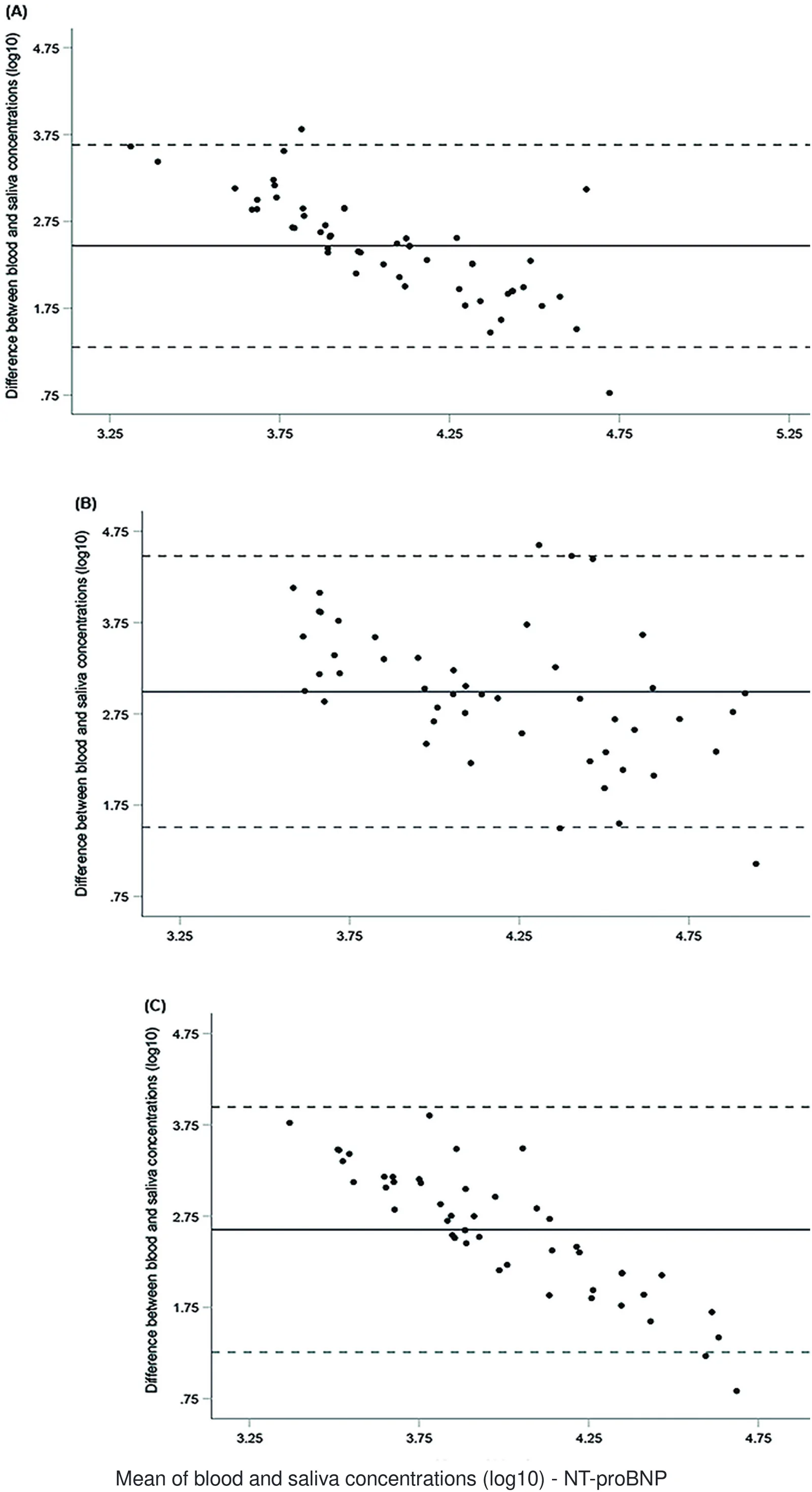
Bland–Altman plots showing agreement between serum and salivary hs-CRP concentrations at baseline (A), 3 months (B), and 6 months (C).

Bland–Altman analyses comparing salivary and serum concentrations of NT-proBNP, cardiac troponin, and hs-CRP across baseline, 3-month, and 6-month follow-up are presented in Figs 4–6. For NT-proBNP and troponin, the plots consistently showed wide dispersion of differences across the range of mean concentrations, with mean bias displaced from zero and broad limits of agreement at all time points, indicating substantial variability between the two biological matrices. In contrast, hs-CRP exhibited mean differences closer to zero and comparatively narrower dispersion; however, the limits of agreement remained wide, and marked inter-individual variability persisted. Overall, visual inspection of the Bland–Altman plots indicate limited agreement between salivary and serum measurements for all three biomarkers throughout follow-up, with no clear improvement over time (Fig 4).

The difference between paired measurements (serum minus saliva) is plotted against their mean on the log10 scale. The solid horizontal line represents the mean difference (bias), and the dashed lines represent the 95% limits of agreement (mean difference ± 1.96 standard deviations) (Fig 5).

Differences between paired measurements (serum minus saliva) are plotted against their mean on the log10 scale. The solid line denotes the mean bias, and dashed lines indicate the 95% limits of agreement (Fig 6).

The difference between serum and salivary measurements is plotted against their mean on the log10 scale. The solid line represents the mean difference (bias), and the dashed lines represent the 95% limits of agreement.

Potential effect modification by age was evaluated using linear regression models including an interaction term between salivary biomarker concentrations and age. No statistically significant interactions were observed for any biomarker at any time point, and interaction coefficients were small, with 95% confidence intervals including the null value. Additional analyses stratified by sex did not reveal consistent or reproducible differences in the relationship between salivary and serum biomarker concentrations.

## Discussion

This study investigated the presence and potential clinical relevance of key cardiac and inflammatory biomarkers in patients with HF through the parallel assessment of NT-proBNP, cardiac troponin, and hs-CRP in paired saliva and blood samples. By examining the detectability of these biomarkers in whole saliva and comparing salivary concentrations with corresponding serum levels over time, we sought to understand the extent to which saliva reflects systemic biomarker dynamics.

Based on our initial hypothesis, we proposed that NT-proBNP, troponin, and hs-CRP would be detectable in the saliva of patients with HF. Our results confirm this assumption, demonstrating that all three biomarkers were consistently detectable in whole saliva.

Notably, these findings are consistent with previous studies demonstrating the detectability of cardiac and inflammatory biomarkers in both saliva and serum across different populations and clinical contexts. Overall, this body of evidence reinforces the biological plausibility of salivary biomarker assessment and corroborates the findings of the present study [13–17].

NT-proBNP has been shown to be detectable in saliva, with significantly higher salivary levels in patients with heart failure than in healthy controls, suggesting potential diagnostic value [18].

The study conducted by Dekker et al. aimed to (1) determine the correlations between salivary and serum levels of BNP, CRP, IL-6, and IL-10 and (2) assess salivary–serum agreement using the Bland–Altman method. There was a moderate-to-strong correlation for serum–salivary CRP, weak correlation for serum–salivary IL-6, and no correlations for serum–salivary BNP and IL-10. The Bland–Altman test showed good salivary–serum agreement for all biomarkers, but as serum concentrations rose, salivary measures underestimated serum levels [14]. The relatively poor salivary–serum agreement observed in the present study contrasts with the better agreement reported by Dekker et al. This discrepancy may be explained by differences in the analytical platforms used to quantify salivary biomarkers, by the specific population evaluated here (heart failure with established periodontitis, which may independently alter salivary composition), and by the fact that Dekker et al. also did not pre-specify clinically acceptable limits of agreement, which may have led to a more permissive qualitative interpretation of their Bland–Altman results.

It has been reported that, although salivary NT-proBNP does not demonstrate diagnostic performance, an association between salivary and serum NT-proBNP levels was observed in patients with acute decompensated heart failure, suggesting that salivary NT-proBNP may have potential value for monitoring treatment response using serial measurements during hospitalization or in the early post-discharge period [19].

More recently, it has been reported that salivary cardiac troponin does not correlate with serum levels and shows limited discriminatory performance for myocardial injury, underscoring the limitations of salivary troponin as a surrogate marker of systemic concentrations in clinical settings [17].

Our second hypothesis was that, in patients with HF, salivary concentrations of NT-proBNP, troponin, and hs-CRP would correlate with their respective serum levels. However, this hypothesis was not supported by our findings. No statistically significant correlations were observed between salivary and serum levels of these biomarkers across the evaluated time points.

According to our data (Figs 4-6), Bland–Altman analysis demonstrated that, for the NT-proBNP biomarker, salivary measurements did not show clinically acceptable agreement with serum measurements at any of the evaluated time points, indicating that, in the present study, saliva did not demonstrate interchangeability with blood for this biomarker. Similarly, salivary troponin did not demonstrate acceptable agreement with serum troponin at any of the assessed time points. It should be noted that this determination was based on qualitative visual assessment of the Bland–Altman plots rather than on a pre-specified, clinically validated agreement threshold.

In contrast, hs-CRP showed a mean bias close to zero between salivary and serum measurements across all evaluated time points, with moderate limits of agreement and no consistent proportional bias. These findings suggest relatively better agreement for hs-CRP compared with the other biomarkers assessed, although inter-individual variability remains and limits full interchangeability between the methods.

Overall, our results indicate that saliva did not demonstrate sufficient agreement to replace blood sampling for the quantification of the evaluated biomarkers, particularly NT-proBNP and troponin.

We also explored whether demographic variables, including age and sex, influenced the relationship between salivary and serum biomarker concentrations. Overall, no consistent pattern of association was observed, suggesting that the variability between salivary and serum measurements in our cohort was not substantially driven by these factors.

Strengths and limitations of the present study should be explicitly acknowledged. The eligibility criteria were deliberately restrictive, limiting enrollment to patients with HFmrEF/HFrEF and to those with stage II or III periodontitis, in order to reduce phenotypic heterogeneity and strengthen the internal validity of the salivary–serum comparison; this same design choice, however, inherently limits the external validity and generalizability of the findings, as detailed below. In the present study, we restricted inclusion to patients with HFmrEF and HFrEF in order to minimize phenotypic heterogeneity in a relatively small exploratory sample focused on biomarker agreement between saliva and sérum [4,7]. This design choice allowed a more homogeneous assessment of patients with impaired systolic function. However, patients with HFpEF were not represented, and this should be considered when interpreting the findings. Given that HFpEF is a prevalent and growing heart failure phenotype worldwide, the present results should not be extrapolated to the overall heart failure population. This aspect impairs study’s external validity. Although the sample size was originally calculated for the primary objective of the clinical study from which this cohort was derived, the number of participants included in the present analysis may still have limited statistical power to detect subtle associations between salivary and serum biomarkers. In addition, biological variability inherent to saliva may have contributed to the heterogeneity observed. Furthermore, despite the use of standardized procedures, pre-analytical and analytical challenges related to salivary assays remain relevant in this field. Moreover, this was a single-center study conducted at a single tertiary care hospital, which may further restrict the generalizability of the findings to other populations and healthcare settings.

Additionally, the inclusion of patients with stage II or III periodontitis as an eligibility criterion – inherited from the design of the parent interventional study – represents a further limitation on external validity. Periodontal inflammation increases the permeability of the sulcular epithelium and alters the protein composition of whole saliva, which may have influenced salivary biomarker concentrations independently of systemic cardiovascular status. Accordingly, the present findings should not be directly extrapolated to heart failure patients without established periodontal disease. This specific clinical combination – heart failure with reduced or mildly reduced ejection fraction in the presence of established periodontitis – had not previously been investigated with respect to salivary-serum biomarker agreement, which underscores the exploratory nature of this work and the need for future studies in broader heart failure populations. Notably, during the screening phase of the parent study, a substantial number of patients were excluded because they were fully edentulous, which precluded periodontal characterization and further highlights the need to study salivary biomarker behavior across the full spectrum of oral health conditions in heart failure patients. Finally, the present analysis did not pre-specify clinically acceptable limits of agreement prior to conducting the Bland–Altman analyses, which is a recommended practice in formal method comparison studies. Given that this was a secondary exploratory analysis in a previously uninvestigated population, an evidence-based threshold could not be established a priori. This limitation should be considered when interpreting the conclusions regarding clinical acceptability of agreement.

Clinically, our findings indicate that salivary NT-proBNP and troponin do not show sufficient agreement with serum concentrations to support their use as substitutes for blood-based assessment in HF. In contrast, hs-CRP demonstrated relatively better agreement, although variability still limits its clinical interchangeability. These results highlight the need for caution when interpreting salivary biomarkers and underscore that serum remains the reference standard for cardiovascular biomarker assessment in heart failure. Notwithstanding these findings, saliva remains a biologically rich and non-invasive matrix, and its potential role in cardiovascular biomarker research warrants continued investigation, particularly in populations and clinical settings not yet explored.

Future studies should prioritize larger multicenter, and more representative samples, including patients with HFpEF and a broader range of periodontal health conditions, as well as the evaluation of different analytical platforms and laboratory approaches, to improve the understanding of variability in salivary biomarkers. The assessment of additional biomarkers may also help identify more promising targets for the application of saliva in specific cardiovascular practice settings.

## Conclusions

In conclusion, NT-proBNP, troponin, and hs-CRP were detectable in saliva from patients with HF; however, salivary concentrations showed insufficient agreement with serum levels for NT-proBNP and troponin, limiting their applicability as substitutes for blood-based assessment. Although hs-CRP demonstrated relatively better agreement between saliva and serum, the observed variability still restricts its clinical interchangeability. Overall, these findings indicate that, under the conditions evaluated, serum remains the reference standard for cardiovascular biomarker assessment in HF, and saliva should be regarded as a matrix warranting further investigation rather than a current clinical alternative. These conclusions should be interpreted with caution, given the restricted generalizability of the study population.
