# Supplementary material for: Salivary and serum levels of NT-proBNP, troponin, and high-sensitivity C-reactive protein in patients with heart failure
Source: PLoS One. 2026 Sep 18;21(9):e0357206. doi: 10.1371/journal.pone.0357206 (PMC13588527; doi:10.1371/journal.pone.0357206)
Supplement: Supplementary file 1 [file pone.0357206.s002.docx]

Responses to Reviewers

PONE-D-26-06014

Salivary and Serum Levels of NT-proBNP, Troponin, and High-Sensitivity C-Reactive Protein in Patients with Heart Failure

Dear Reviewers,

We would like to express our appreciation to both reviewers for their thoughtful and valuable suggestions. Their comments were highly relevant and helped us improve the clarity, transparency, and overall quality of the manuscript.

In response to the comments from the editors and reviewers, we carefully revised the manuscript and addressed all points raised. In this file you will find a detailed point-by-point response. In addition, we have submitted both a revised version with track changes and a clean revised version of the manuscript.

# Reviewer 1

## 1 – The introduction looks redundant and should be extensively revised and shortened. Lines from 68 to 81 are not necessary. It should be shortened, focusing on the research topic.

**Response:** We thank the reviewer for this valuable suggestion. The Introduction has been carefully revised and substantially shortened to improve clarity and focus on the main research topic. As recommended, the content corresponding to lines 68–81 has been removed, and redundant sections were edited to provide a more concise background for the study.

## 2 – The cohort is relatively small and recruited over a very short time window, raising concerns about potential selection bias and representativeness. It should be clearly discussed.

**Response:** We thank the reviewer for this important comment. The relatively short recruitment period is explained by the fact that the present study represents a secondary analysis of participants enrolled in a clinical research program involving patients with heart failure. Before the interventional phase, an observational study had already been approved and initiated in 2023, which allowed the research team to identify and follow eligible patients within the same clinical setting. Specifically, the observational study was registered in the Brazilian public research registry (CAAE: 63946522.7.0000.5501; approval number 5.708.195). Subsequently, the interventional study was approved under a second protocol (CAAE: 7662.1623.3.0000.5501; approval number 6.651.994). Because participants were already being screened and followed within this research framework, recruitment for the interventional phase occurred over a shorter time window. This information has been clarified in the revised manuscript.

## 3 – Key clinical characteristics are insufficiently detailed (e.g., NYHA class, renal function, rhythm, comorbidities, medication, clinical stability), all of which may influence circulating and salivary biomarker levels. It should be clearly stated.

**Response:** We thank the reviewer for this important comment. Additional information on the clinical profile of the participants has been included in the revised manuscript. All participants were under guideline-directed medical therapy for heart failure with reduced or mildly reduced ejection fraction, according to contemporary international recommendations. Optimized pharmacological treatment included a beta-blocker, inhibition of the renin–angiotensin system (angiotensin-converting enzyme inhibitor, angiotensin receptor blocker, or angiotensin receptor–neprilysin inhibitor), a mineralocorticoid receptor antagonist, and a sodium–glucose cotransporter-2 inhibitor when clinically indicated and tolerated. Diuretics were prescribed when necessary for the management of congestion.

Functional status was assessed using the Kansas City Cardiomyopathy Questionnaire (KCCQ), a validated disease-specific instrument that evaluates symptoms, physical limitation, quality of life, and social limitation in patients with heart failure. Because KCCQ scores are strongly associated with functional status and NYHA class, this measure was used as a complementary indicator of clinical status. We also clarified that no participant changed KCCQ category during the follow-up period, supporting clinical stability during the study.

In addition, the revised manuscript now reports cardiac rhythm distribution: 37 patients (71.2%) had sinus rhythm, 13 (25.0%) had atrial fibrillation, and 2 (3.8%) had atrial fibrillation with pacemaker.

**References:**

- Green CP, Porter CB, Bresnahan DR, Spertus JA. Development and evaluation of the Kansas City Cardiomyopathy Questionnaire: a new health status measure for heart failure. J Am Coll Cardiol. 2000;35(5):1245-1255. doi:10.1016/S0735-1097(00)00531-3.
- Spertus JA, Jones PG. Development and validation of a short version of the Kansas City Cardiomyopathy Questionnaire. Circ Cardiovasc Qual Outcomes. 2015;8(5):469-476. doi:10.1161/CIRCOUTCOMES.115.001958.
- Spertus JA, Jones PG, Sandhu AT, Arnold SV. Interpreting the Kansas City Cardiomyopathy Questionnaire in clinical trials and clinical care: JACC State-of-the-Art Review. J Am Coll Cardiol. 2020;76(20):2379-2390. doi:10.1016/j.jacc.2020.09.542.

## 4 – Exclusion of HFpEF patients should be better justified and explicitly acknowledged as a limitation to generalizability.

**Response:** We thank both reviewers for these important and complementary comments. We have clarified throughout the revised manuscript that the study population included only patients with heart failure with mildly reduced ejection fraction (HFmrEF; left ventricular ejection fraction [LVEF] 41–49%) and heart failure with reduced ejection fraction (HFrEF; LVEF ≤40%), according to current guideline-based classification. Patients with heart failure with preserved ejection fraction (HFpEF; LVEF ≥50%) were not included.

This restriction was intentional and was adopted to reduce phenotypic and pathophysiological heterogeneity in a relatively small exploratory biomarker study. By limiting the sample to HFmrEF and HFrEF, we aimed to provide a more homogeneous assessment of saliva–serum biomarker agreement in patients with impaired systolic function. We agree, however, that the exclusion of HFpEF limits the external validity and generalizability of our findings. HFpEF is a prevalent and growing heart failure phenotype worldwide, and therefore our results should not be extrapolated to the overall heart failure population, particularly not to patients with HFpEF.

Accordingly, we revised the Abstract, Methods, and Discussion to (i) explicitly define the included heart failure phenotypes, (ii) correct the terminology previously used for the combined HFmrEF/HFrEF group, and (iii) acknowledge the exclusion of HFpEF as a study limitation.

**References:**

- Heidenreich PA, Bozkurt B, Aguilar D, Allen LA, Byun JJ, Colvin MM, et al. 2022 AHA/ACC/HFSA guideline for the management of heart failure. Circulation. 2022;145(18):e895-e1032. doi:10.1161/CIR.0000000000001063.
- McDonagh TA, Metra M, Adamo M, Gardner RS, Baumbach A, Böhm M, et al. 2023 focused update of the 2021 ESC Guidelines for the diagnosis and treatment of acute and chronic heart failure. Eur Heart J. 2023;44(37):3627-3639. doi:10.1093/eurheartj/ehad195.

## 5 – Standardization of collection time and use of unstimulated saliva are strengths. However, no information is provided on oral health status (periodontal disease, gingival bleeding), salivary flow, xerostomia, smoking, or oral hygiene, all of which may significantly affect salivary composition and concentrations. Additional clarification is needed.

**Response:** We thank the reviewer for this important observation. The revised manuscript now clarifies that current use of tobacco and/or electronic cigarettes, as well as reduced salivary flow, were considered exclusion criteria. Periodontitis was also explicitly described among the clinical characteristics of the study population. In addition, mean salivary flow, plaque index, and gingival index had been added to the Results section.

## 6 – The discussion section seems redundant as well, and I would shorten it, focusing on the main messages.

**Response:** We thank the reviewer for this valuable suggestion. The Discussion section was revised and streamlined to reduce redundancy and improve clarity. We shortened repetitive passages and refocused the text on the main findings of the study. In addition, we strengthened the limitations section by explicitly addressing the restricted heart failure phenotypes included (HFmrEF/HFrEF), the exclusion of HFpEF, and the relatively small sample size, all of which may limit the generalizability of the findings.

## 7 – Tables are clear, but it should be explicitly stated throughout when values are log-transformed.

**Response:** We agree with the reviewer’s suggestion. The manuscript has been revised to consistently indicate that biomarker concentrations were analyzed and presented on the log10 scale, including in the Results section and in the relevant table and figure descriptions.

## 8 – I would add some nice figures showing the biomarker trend across each timepoint with values and confidence intervals.

**Response:** We appreciate this valuable suggestion. New figures 1 to 3 were added to illustrate the temporal trends of NT-proBNP, troponin, and hs-CRP across the three evaluation time points (baseline, 3 months, and 6 months). These figures show mean values and corresponding 95% confidence intervals for both serum and saliva, allowing clearer visualization of temporal patterns and variability. Original figures 1 to 3 had been renumbered as figures 4 to 6 (Bland-Altman data).

# Reviewer 2

## 1 – Introduction is extremely long and must be condensed in one page by focusing on the main topic of the paper.

**Response:** We thank the reviewer for this valuable suggestion. The Introduction has been carefully revised and substantially shortened to improve clarity and focus on the main research topic.

## 2 – IRB approval number must be provided.

**Response:** We thank the reviewer for this comment. The revised manuscript now clearly reports both ethics approvals related to the research framework from which this analysis was derived: the observational study (CAAE: 63946522.7.0000.5501; approval number 5.708.195) and the subsequent interventional study (CAAE: 7662.1623.3.0000.5501; approval number 6.651.994).

## 3 – Not clear whether patients with HFrEF and/or HFmrEF were included. Similarly, why did authors include only a portion of HF patients? Why excluding HFpEF patients, who are progressively increasing all around the world?

**Response:** We thank both reviewers for these important and complementary comments. We have clarified throughout the revised manuscript that the study population included only patients with heart failure with mildly reduced ejection fraction (HFmrEF; left ventricular ejection fraction [LVEF] 41–49%) and heart failure with reduced ejection fraction (HFrEF; LVEF ≤40%), according to current guideline-based classification. Patients with heart failure with preserved ejection fraction (HFpEF; LVEF ≥50%) were not included.

This restriction was intentional and was adopted to reduce phenotypic and pathophysiological heterogeneity in a relatively small exploratory biomarker study. By limiting the sample to HFmrEF and HFrEF, we aimed to provide a more homogeneous assessment of saliva–serum biomarker agreement in patients with impaired systolic function. We agree, however, that the exclusion of HFpEF limits the external validity and generalizability of our findings. HFpEF is a prevalent and growing heart failure phenotype worldwide, and therefore our results should not be extrapolated to the overall heart failure population, particularly not to patients with HFpEF.

Accordingly, we revised the Abstract, Methods, and Discussion to (i) explicitly define the included heart failure phenotypes, (ii) correct the terminology previously used for the combined HFmrEF/HFrEF group, and (iii) acknowledge the exclusion of HFpEF as a study limitation.

**References:**

- Heidenreich PA, Bozkurt B, Aguilar D, Allen LA, Byun JJ, Colvin MM, et al. 2022 AHA/ACC/HFSA guideline for the management of heart failure. Circulation. 2022;145(18):e895-e1032. doi:10.1161/CIR.0000000000001063.
- McDonagh TA, Metra M, Adamo M, Gardner RS, Baumbach A, Böhm M, et al. 2023 focused update of the 2021 ESC Guidelines for the diagnosis and treatment of acute and chronic heart failure. Eur Heart J. 2023;44(37):3627-3639. doi:10.1093/eurheartj/ehad195.

## 4 – Please correct “gender” with “sex”.

**Response:** We acknowledge the reviewer’s suggestion. The term “gender” has been replaced with “sex” throughout the manuscript.

## 5 – Additional information about ELISA kits must be provided (manufacturer, intra- and inter-assay coefficients).

**Response:** We agree with the reviewer’s suggestion. Additional methodological details regarding the ELISA kits, including manufacturer information and the intra-assay and inter-assay coefficients of variation, have been added to the Methods section to improve clarity and reproducibility.

## 6 – “Descriptive analyses were performed for biomarker variables according to biomarker type, biological matrix (blood and saliva), and sampling time” --> what does this mean?

**Response:** We appreciate the reviewer’s request for clarification. In this context, descriptive analyses refer to the summary of biomarker concentrations according to biomarker type (NT-proBNP, troponin, and hs-CRP), biological matrix (serum and saliva), and follow-up time point (baseline, 3 months, and 6 months). To improve clarity, this description has been revised in the Statistical Analysis section.

## 7 – A sample size is highly recommended to better evaluate the results.

**Response:** We thank the reviewer for this important comment. A sample size calculation was performed during the planning phase of the parent clinical study from which the present dataset was derived. This calculation was originally based on the primary objective of that study, which assessed the effects of periodontal treatment on clinical parameters and systemic biomarkers in patients with heart failure.

**Response:** The estimation was based on expected differences in BNP and NT-proBNP levels, assuming a minimum detectable difference of 10%. Based on this calculation, and including a 10% safety margin, the estimated sample size was approximately 25 participants per group (treatment and control). The present manuscript represents a secondary analysis of this cohort, focusing specifically on the agreement between salivary and serum biomarker measurements. Therefore, all available participants with heart failure were analyzed together (n = 52), irrespective of their original allocation. This clarification has been added to the Methods section.

## 8 – I would recommend authors to provide evidence supporting internal validity of the results.

**Response:** We thank the reviewer for this important suggestion. Several methodological procedures were implemented to strengthen the internal validity of the study. First, the study followed a prospective longitudinal design with standardized data collection at three predefined time points. Blood and saliva samples were collected using standardized protocols, and saliva collection was performed under controlled conditions within a fixed morning time window to reduce potential circadian variability.

Second, laboratory analyses were conducted using validated assays and standardized procedures according to the manufacturers’ recommendations. Samples were processed and stored under controlled conditions (−80°C) to minimize pre-analytical variability. In addition, all ELISA assays were performed in duplicate, and the mean of duplicate measurements was used for statistical analyses to improve analytical precision and reliability.

Finally, appropriate statistical approaches were applied according to the characteristics of the data, including log transformation of biomarker concentrations, non-parametric correlation analyses with bootstrap confidence intervals, and Bland–Altman analysis to evaluate agreement between biological matrices. These points have now been more explicitly highlighted in the revised manuscript.

## 9 – I would recommend authors to provide additional information about comorbidities of the patients included in the study. Also, including treatments during the study period would represent an important aspect to be added in the manuscript.

**Response:** We thank the reviewer for this important comment. Additional information regarding heart failure treatment has been included in the Methods section. All participants were under guideline-directed medical therapy for heart failure with reduced or mildly reduced ejection fraction, according to contemporary international recommendations. Optimized pharmacological treatment included a beta-blocker, inhibition of the renin–angiotensin system (angiotensin-converting enzyme inhibitor, angiotensin receptor blocker, or angiotensin receptor–neprilysin inhibitor), a mineralocorticoid receptor antagonist, and a sodium–glucose cotransporter-2 inhibitor when clinically indicated and tolerated. Diuretics were prescribed when necessary for congestion management.

We also clarified the clinical stability of the sample through KCCQ-based functional assessment and added cardiac rhythm distribution to the revised manuscript: 37 patients (71.2%) had sinus rhythm, 13 (25.0%) had atrial fibrillation, and 2 (3.8%) had atrial fibrillation with pacemaker.

- Green CP, Porter CB, Bresnahan DR, Spertus JA. Development and evaluation of the Kansas City Cardiomyopathy Questionnaire: a new health status measure for heart failure. J Am Coll Cardiol. 2000;35(5):1245-1255. doi:10.1016/S0735-1097(00)00531-3.
- Spertus JA, Jones PG. Development and validation of a short version of the Kansas City Cardiomyopathy Questionnaire. Circ Cardiovasc Qual Outcomes. 2015;8(5):469-476. doi:10.1161/CIRCOUTCOMES.115.001958.
- Spertus JA, Jones PG, Sandhu AT, Arnold SV. Interpreting the Kansas City Cardiomyopathy Questionnaire in clinical trials and clinical care: JACC State-of-the-Art Review. J Am Coll Cardiol. 2020;76(20):2379-2390. doi:10.1016/j.jacc.2020.09.542.

## 10 – Discussion is extremely long compared to the content of the paper. Please revise and shorten it accordingly.

**Response:** We thank the reviewer for this valuable suggestion. The Discussion has been revised for conciseness and clarity, with removal of repetitive statements and greater focus on the central findings and their interpretation. The limitations subsection was also expanded to explicitly address the inclusion of only HFmrEF/HFrEF patients, the exclusion of HFpEF, and the relatively small sample size.
