# Supplementary material for: Salivary and serum levels of NT-proBNP, troponin, and high-sensitivity C-reactive protein in patients with heart failure
Source: PLoS One. 2026 Sep 18;21(9):e0357206. doi: 10.1371/journal.pone.0357206 (PMC13588527; doi:10.1371/journal.pone.0357206)
Supplement: Supplementary file 2 [file pone.0357206.s003.docx]

***Answering letter***

PONE-D-26-06014

Salivary and Serum Levels of NT-proBNP, Troponin, and High-Sensitivity C-Reactive Protein in Patients with Heart Failure

Dear Reviewers,

In response to all points raised, we have carefully revised the manuscript. A detailed point-by-point response is provided below. We have also submitted a revised version with track changes and a clean revised version of the manuscript.

## 1 – Overall, this is a very interesting study. [...] One comment I have is regarding the conclusion that saliva should be considered a “complementary matrix” for cardiovascular biomarker assessment. The data do not clearly support this framing. All three biomarkers showed weak, non-significant correlations between salivary and serum concentrations at all time points, and Bland-Altman analyses demonstrated clinically unacceptable agreement for NT-proBNP and troponin. While hs-CRP showed a mean bias closer to zero, the limits of agreement remained wide and inter-individual variability was substantial. Given these findings, it is unclear in what clinical scenario salivary measurements would meaningfully complement serum-based assessment. If you believe that salivary testing can complement serum testing, you should either provide a specific, clinically grounded example of how salivary biomarkers could add value alongside serum measurements, or adjust the language to more accurately reflect the findings – characterizing saliva as a matrix warranting further investigation rather than a current complementary tool.

**Response:** We fully agree that the original framing of saliva as a “complementary matrix” was not adequately supported by the data and may have overstated the clinical utility of salivary biomarker measurement in this context. Accordingly, we have revised the language throughout the manuscript – in the Abstract, Discussion, and Conclusions – to characterize saliva as a “matrix warranting further investigation” rather than a current complementary tool. We have also added a statement in the Discussion acknowledging that, despite the limited agreement observed in the present study, saliva remains a biologically rich and non-invasive matrix whose potential for cardiovascular biomarker assessment merits continued research, particularly in populations and clinical conditions not yet explored. Furthermore, the Conclusions now explicitly state that serum remains the reference standard for the assessment of NT-proBNP, troponin, and hs-CRP in patients with heart failure, based on the data presented.

## 2 – Inclusion of patients with Stage II or III periodontitis as an eligibility criterion deserves mention as a potential limitation on external validity, as periodontal disease may influence salivary composition and biomarker concentrations. While this was driven by the design of the parent study, it restricts the applicability of findings to the broader heart failure population. This potentially opens up the door for having the study repeated in a population without periodontal disease.

**Response:** We fully agree and have added this limitation explicitly to the Discussion. The inclusion of patients with stage II or III periodontitis – a criterion inherited from the parent interventional study – may have influenced salivary composition and biomarker concentrations, given that periodontal inflammation increases the permeability of the sulcular epithelium and alters the protein content of whole saliva. Accordingly, the present findings cannot be directly extrapolated to the broader population of heart failure patients without established periodontal disease.

We also wish to highlight that this specific combination, heart failure with reduced or mildly reduced ejection fraction in the presence of established periodontitis, had not previously been investigated with respect to salivary-serum biomarker agreement using the Bland-Altman method. This constitutes a key aspect of the originality and exploratory nature of the present study. Notably, during the screening phase of the parent study, a substantial number of patients with heart failure were excluded because they were fully edentulous, which precluded periodontal characterization. This observation underscores the clinical complexity of this population and further highlights the need for future studies exploring salivary biomarker behavior across a broader spectrum of oral health conditions in heart failure patients, including those without periodontal disease and those who are edentulous.

## 3 – One methodological gap worth noting is the absence of pre-specified, clinically acceptable limits of agreement prior to conducting the Bland-Altman analysis. In a measurement agreement study, defining acceptable thresholds prior to strengthens the interpretability of results. Without this, the determination that agreement is “clinically unacceptable” for NT-proBNP and troponin – while intuitively supported by the data – rests on post-hoc visual and qualitative judgment rather than a prospectively defined criterion.

**Response:** We recognize that the pre-specification of clinically acceptable limits of agreement is a recommended practice in formal method comparison studies, and we agree that its absence represents a limitation of the present analysis.

It is important to note, however, that the present work is a secondary analysis derived from a parent interventional study whose primary objective was to evaluate the effects of periodontal treatment on systemic biomarkers in patients with heart failure. The assessment of salivary-serum biomarker agreement was not the primary endpoint, and the study population – patients with heart failure and established stage II or III periodontitis – represents a clinical combination with scarce information. Therefore, the definition of a clinically acceptable threshold a priori would have lacked an adequate empirical basis.

## Additional note – Public data repository

We would like to take this opportunity to reaffirm that all data underlying the findings of this study are publicly available in the NDEx repository under the dataset “heart failure dataset network,” accessible at https://doi.org/10.18119/N9JC8K. This information is included in the Data Availability statement of the manuscript. We apologize if this was not readily apparent in the previous submission and hope the present clarification addresses any remaining concern.
