# Supplementary material for: Salivary and serum levels of NT-proBNP, troponin, and high-sensitivity C-reactive protein in patients with heart failure
Source: PLoS One. 2026 Sep 18;21(9):e0357206. doi: 10.1371/journal.pone.0357206 (PMC13588527; doi:10.1371/journal.pone.0357206)
Supplement: Supplementary file 3 [file pone.0357206.s004.docx]

**Answering letter**

PONE-D-26-06014R2

Salivary and Serum Levels of NT-proBNP, Troponin, and High-Sensitivity C-Reactive Protein in Patients with Heart Failure

Dear Editor,

We thank you for your positive evaluation of our revised manuscript and for the opportunity to submit a further revision. We greatly appreciate your assessment that the previous reviewer comments were thoughtfully addressed and that the overall quality of the paper has improved substantially. Below we provide a point-by-point response to each of the three remaining points raised in your letter, as well as to the journal's general requirements regarding the reference list. All corresponding changes are highlighted in the revised manuscript with track changes.

**1 – Clarifying the interpretation of your findings.**

Response: We thank the Academic Editor for this comment. While revising the Discussion, we identified and corrected two citation errors that had inadvertently attributed findings on salivary NT-proBNP to reference [17], a study that specifically addresses salivary troponin. The sentence describing higher salivary NT-proBNP levels in heart failure patients compared with healthy controls now correctly cites Foo et al. (reference [18]), and the sentence describing the association between salivary and serum NT-proBNP levels in acute decompensated heart failure now correctly cites Rammos et al. (reference [19]).

We also added a sentence reconciling why the limited salivary–serum agreement observed in the present study contrasts with the better agreement previously reported by Dekker et al. [14], noting as possible explanations the different analytical platforms used, the specific population evaluated here (heart failure with established periodontitis, which may independently alter salivary composition), and the fact that Dekker et al. also did not pre-specify clinically acceptable limits of agreement (Discussion).

Finally, we added a sentence at the point where salivary NT-proBNP and troponin agreement is first classified as clinically unacceptable, clarifying that this determination reflects qualitative visual assessment of the Bland–Altman plots rather than a pre-specified threshold.

**2 – Moderating some of the conclusions to better align with the presented data.**

Response: We thank the Academic Editor for this observation. We removed a duplicated sentence in the Limitations paragraph that had been left over from the previous revision, and we revised language in the Discussion that was more absolute than warranted by our data. Specifically, the statement “saliva cannot be considered an interchangeable matrix with blood for this biomarker” was revised to “in the present study, saliva did not demonstrate interchangeability with blood for this biomarker,” aligning the tone of this sentence with the more conditional language already used in the Abstract and Conclusions (“under the conditions evaluated”).

We also added a concluding sentence to the Conclusions section, explicitly stating that our conclusions should be interpreted with caution given the restricted generalizability of the study population.

**3 – Explicitly acknowledging methodological limitations, including the restricted external validity of the study population and the absence of pre-specified clinically acceptable limits of agreement.**

Response: We agree that these limitations warranted more explicit treatment and have expanded the Limitations paragraph accordingly.

We added a sentence at the beginning of the paragraph explicitly framing our restrictive eligibility criteria (HFmrEF/HFrEF and stage II or III periodontitis) as a deliberate design choice made to reduce phenotypic heterogeneity and strengthen the internal validity of the salivary–serum comparison, at the cost of external validity and generalizability (Discussion). This framing extends, to the periodontal eligibility criterion, the same rationale previously accepted by the reviewer with respect to our ejection-fraction-based inclusion criteria.

We further acknowledged that this was a single-center study conducted at a single tertiary care hospital, which may additionally restrict the generalizability of our findings to other populations and healthcare settings (Discussion). Accordingly, we expanded our recommendations for future research to explicitly call for multicenter studies enrolling a broader range of patients, including those with HFpEF and varying degrees of periodontal health (Discussion).

Regarding the absence of pre-specified, clinically acceptable limits of agreement, this limitation was already acknowledged in our previous revision (Discussion, Limitations paragraph); we have now additionally flagged it earlier in the Discussion, at the exact point where salivary–serum agreement is first classified as clinically unacceptable, with a cross-reference directing the reader to the Limitations paragraph for full context (Discussion).

**Journal Requirements**

Response regarding the reference list: We have reviewed our reference list in full. As described above, two in-text citation numbers were corrected (references [18] and [19] are now correctly cited instead of [17] in two instances); no references were added to or removed from the list, and no new citations were introduced. We also verified retraction status for all 19 references, with particular attention to the more recent and specialized sources (references 7 and 14–19); we found no evidence that any of the cited articles have been retracted.

We hope that the present revision fully addresses the Academic Editor's comments, and we remain at your disposal for any further clarification.

Sincerely,

José Roberto Cortelli

On behalf of all authors
