# Supplementary material for: Salivary and serum levels of NT-proBNP, troponin, and high-sensitivity C-reactive protein in patients with heart failure
Source: PLoS One. 2026 Sep 18;21(9):e0357206. doi: 10.1371/journal.pone.0357206 (PMC13588527; doi:10.1371/journal.pone.0357206)
Supplement: Peer review history

# Peer review: Decision Letter 0

**DOI:** 10.1371/journal.pone.0357206.r001

**Authors:** Michele Golino

4 Mar 2026

Dear Dr. cortelli,

Thank you for submitting your manuscript to PLOS ONE. After careful consideration, we feel that it has merit but does not fully meet PLOS ONE’s publication criteria as it currently stands. Therefore, we invite you to submit a revised version of the manuscript that addresses the points raised during the review process.

Please submit your revised manuscript by Apr 18 2026 11:59PM. If you will need more time than this to complete your revisions, please reply to this message or contact the journal office at plosone@plos.org. When you’re ready to submit your revision, log on to https://www.editorialmanager.com/pone/ and select the ‘Submissions Needing Revision’ folder to locate your manuscript file.

- A letter that responds to each point raised by the academic editor and reviewer(s). You should upload this letter as a separate file labeled ‘Response to Reviewers’.
- A marked-up copy of your manuscript that highlights changes made to the original version. You should upload this as a separate file labeled ‘Revised Manuscript with Track Changes’.
- An unmarked version of your revised paper without tracked changes. You should upload this as a separate file labeled ‘Manuscript’.

If you would like to make changes to your financial disclosure, please include your updated statement in your cover letter. Guidelines for resubmitting your figure files are available below the reviewer comments at the end of this letter.

If applicable, we recommend that you deposit your laboratory protocols in protocols.io to enhance the reproducibility of your results. Protocols.io assigns your protocol its own identifier (DOI) so that it can be cited independently in the future. For instructions see: https://journals.plos.org/plosone/s/submission-guidelines#loc-laboratory-protocols. Additionally, PLOS ONE offers an option for publishing peer-reviewed Lab Protocol articles, which describe protocols hosted on protocols.io. Read more information on sharing protocols at https://plos.org/protocols?utm_medium=editorial-email&utm_source=authorletters&utm_campaign=protocols.

We look forward to receiving your revised manuscript.

Kind regards,

Michele Golino, M.D., Ph.D.

Academic Editor

PLOS One

Journal Requirements:

When submitting your revision, we need you to address these additional requirements.

1. Please ensure that your manuscript meets PLOS ONE’s style requirements, including those for file naming. The PLOS ONE style templates can be found at

https://journals.plos.org/plosone/s/file?id=wjVg/PLOSOne_formatting_sample_main_body.pdf and

https://journals.plos.org/plosone/s/file?id=ba62/PLOSOne_formatting_sample_title_authors_affiliations.pdf

2. Please provide additional details regarding participant consent. In the ethics statement in the Methods and online submission information, please ensure that you have specified (1) whether consent was informed and (2) what type you obtained (for instance, written or verbal, and if verbal, how it was documented and witnessed). If your study included minors, state whether you obtained consent from parents or guardians. If the need for consent was waived by the ethics committee, please include this information.

If you are reporting a retrospective study of medical records or archived samples, please ensure that you have discussed whether all data were fully anonymized before you accessed them and/or whether the IRB or ethics committee waived the requirement for informed consent. If patients provided informed written consent to have data from their medical records used in research, please include this information.

3. Thank you for stating the following financial disclosure:

“This study was supported by the São Paulo Research Foundation (FAPESP) (grant no. 2023/07458-4, awarded to JRC) and by the National Council for Scientific and Technological Development (CNPq) (grant no. 306161/2023-0, awarded to JRC).”

Please state what role the funders took in the study.  If the funders had no role, please state: "The funders had no role in study design, data collection and analysis, decision to publish, or preparation of the manuscript."

If this statement is not correct you must amend it as needed.

Please include this amended Role of Funder statement in your cover letter; we will change the online submission form on your behalf.

4. Thank you for uploading your study’s underlying data set. Unfortunately, the repository you have noted in your Data Availability statement does not qualify as an acceptable data repository according to PLOS’s standards.

At this time, please upload the minimal data set necessary to replicate your study’s findings to a stable, public repository (such as figshare or Dryad) and provide us with the relevant URLs, DOIs, or accession numbers that may be used to access these data. For a list of recommended repositories and additional information on PLOS standards for data deposition, please see https://journals.plos.org/plosone/s/recommended-repositories.

5. We note that you have included the phrase “data not shown” in your manuscript. Unfortunately, this does not meet our data sharing requirements. PLOS does not permit references to inaccessible data. We require that authors provide all relevant data within the paper, Supporting Information files, or in an acceptable, public repository. Please add a citation to support this phrase or upload the data that corresponds with these findings to a stable repository (such as Figshare or Dryad) and provide and URLs, DOIs, or accession numbers that may be used to access these data. Or, if the data are not a core part of the research being presented in your study, we ask that you remove the phrase that refers to these data.

6. If the reviewer comments include a recommendation to cite specific previously published works, please review and evaluate these publications to determine whether they are relevant and should be cited. There is no requirement to cite these works unless the editor has indicated otherwise.

Additional Editor Comments:

Dear Author,

Please try to address Reviewers’ comments.

[Note: HTML markup is below. Please do not edit.]

Reviewers’ comments:

Reviewer’s Responses to Questions

**Comments to the Author**

1. Is the manuscript technically sound, and do the data support the conclusions?

Reviewer #1: Partly

Reviewer #2: Yes

**********

2. Has the statistical analysis been performed appropriately and rigorously?

Reviewer #1: Yes

Reviewer #2: Yes

**********

3. Have the authors made all data underlying the findings in their manuscript fully available?

The PLOS Data policy

Reviewer #1: No

Reviewer #2: Yes

**********

4. Is the manuscript presented in an intelligible fashion and written in standard English?

Reviewer #1: No

Reviewer #2: Yes

**********

Reviewer #1: Viegas et al., conducted a prospective longitudinal study in 52 patients with heart failure (LVEF <40% or 41–49%), with paired saliva and serum samples collected at baseline, 3, and 6 months to measure NT-proBNP, troponin, and hs-CRP. All biomarkers were detectable in saliva; however, saliva–serum correlations (Spearman) were non-significant. Agreement between matrices, assessed using Bland–Altman analysis on log-transformed data, was poor for NT-proBNP and troponin, showing wide limits of agreement and systematic bias, while hs-CRP showed substantial inter-individual variability. Age and sex did not significantly modify these associations, suggesting that salivary biomarkers are not interchangeable with serum measurements in heart failure monitoring.

I have some comments:

-The introduction looks redundant and should be extensively revised and shortened. Lines from 68 to 81 are not necessary. It should be shortened, focusing on the research topic.

-The cohort is relatively small and recruited over a very short time window, raising concerns about potential selection bias and representativeness. It should be clearly discussed.

-Key clinical characteristics are insufficiently detailed (e.g. NYHA class, renal function, rhythm, comorbidities, medication, clinical stability), all of which may influence circulating and salivary biomarker levels. It should be clearly stated in a dedicated Table.

-Exclusion of HFpEF patients should be better justified and explicitly acknowledged as a limitation to generalizability.

-Standardization of collection time and use of unstimulated saliva are strengths. However, no information is provided on oral health status (periodontal disease, gingival bleeding), salivary flow, xerostomia, smoking, or oral hygiene, all of which may significantly affect salivary composition and concentrations. Additional clarification is needed.

-The discussion section seems redundant as well, and I would shorten it, focusing on the main messages.

-Tables are clear, but it should be explicitly stated throughout when values are log-transformed.

-I would add some nice figures showing the biomarker trend across each timepoint with values and confidence intervals.

Reviewer #2: This paper has evaluated the ability of assessing concentrations of some biomakers in the saliva with the same accuracy as currently done in the blood.

The paper is sound, however some concerns are raised during the review process:

- Introduction is extremely long and must be condensed in one page by focusing on the main topic of the paper.

- IRB approval number must be provided.

- Not clear whether patients with HFrEF and/or HFmrEF were included. Similarly, why did authors include only a portion of HF patients? Why excluding HFpEF patients, who are progressively increasing all around the world?

- Please correct "gender" with "sex".

- Additional information about ELISA kits must be provided (manufacturer, intra- and interassay coefficients).

- "Descriptive analyses were performed for biomarker variables according to biomarker type, biological matrix (blood and saliva), and sampling time" --> what does this mean?

- A sample size is highly recommended to better evaluate the results.

- I would recommend authors to provide evidence supporting internal validity of the results.

- I would recommend authors to provide additional information about comorbidities of the patients included in the study. Also, including treatments during the study period would represent an important aspect to be added in the manuscript.

- Discussion is extremely long compared to the content of the paper. Please revise and shorten it accordingly.

**********

what does this mean?). If published, this will include your full peer review and any attached files.

If you choose “no”, your identity will remain anonymous but your review may still be made public.

**Do you want your identity to be public for this peer review?**  For information about this choice, including consent withdrawal, please see our Privacy Policy

Reviewer #1: No

Reviewer #2: No

**********

[NOTE: If reviewer comments were submitted as an attachment file, they will be attached to this email and accessible via the submission site. Please log into your account, locate the manuscript record, and check for the action link "View Attachments". If this link does not appear, there are no attachment files.]

To ensure your figures meet our technical requirements, please review our figure guidelines: https://journals.plos.org/plosone/s/figures

You may also use PLOS’s free figure tool, NAAS, to help you prepare publication quality figures: https://journals.plos.org/plosone/s/figures#loc-tools-for-figure-preparation.

NAAS will assess whether your figures meet our technical requirements by comparing each figure against our figure specifications.

# Author comment: Author response to Decision Letter 1

**DOI:** 10.1371/journal.pone.0357206.r002

20 Apr 2026

Uploaded in a separate file.

Attachment Submitted filename: 3-APR-2026 Responses to Reviewers.docx

# Peer review: Decision Letter 1

**DOI:** 10.1371/journal.pone.0357206.r003

**Authors:** Michele Golino

20 May 2026

Dear Dr. cortelli,

Please submit your revised manuscript by Jul 04 2026 11:59PM. If you will need more time than this to complete your revisions, please reply to this message or contact the journal office at plosone@plos.org. When you’re ready to submit your revision, log on to https://www.editorialmanager.com/pone/ and select the ‘Submissions Needing Revision’ folder to locate your manuscript file.

- A letter that responds to each point raised by the academic editor and reviewer(s). You should upload this letter as a separate file labeled ‘Response to Reviewers’.
- A marked-up copy of your manuscript that highlights changes made to the original version. You should upload this as a separate file labeled ‘Revised Manuscript with Track Changes’.
- An unmarked version of your revised paper without tracked changes. You should upload this as a separate file labeled ‘Manuscript’.

If you would like to make changes to your financial disclosure, please include your updated statement in your cover letter. Guidelines for resubmitting your figure files are available below the reviewer comments at the end of this letter.

If applicable, we recommend that you deposit your laboratory protocols in protocols.io to enhance the reproducibility of your results. Protocols.io assigns your protocol its own identifier (DOI) so that it can be cited independently in the future. For instructions see: https://journals.plos.org/plosone/s/submission-guidelines#loc-laboratory-protocols. Additionally, PLOS ONE offers an option for publishing peer-reviewed Lab Protocol articles, which describe protocols hosted on protocols.io. Read more information on sharing protocols at https://plos.org/protocols?utm_medium=editorial-email&utm_source=authorletters&utm_campaign=protocols.

As the corresponding author, your ORCID iD is verified in the submission system and will appear in the published article. PLOS supports the use of ORCID, and we encourage all coauthors to register for an ORCID iD and use it as well. Please encourage your coauthors to verify their ORCID iD within the submission system before final acceptance, as unverified ORCID iDs will not appear in the published article. *Only*  the individual author can complete the verification step; PLOS staff *cannot*  verify ORCID iDs on behalf of authors.

We look forward to receiving your revised manuscript.

Kind regards,

Michele Golino, M.D., Ph.D.

Academic Editor

PLOS One

Journal Requirements:

If the reviewer comments include a recommendation to cite specific previously published works, please review and evaluate these publications to determine whether they are relevant and should be cited. There is no requirement to cite these works unless the editor has indicated otherwise.

Please review your reference list to ensure that it is complete and correct. If you have cited papers that have been retracted, please include the rationale for doing so in the manuscript text, or remove these references and replace them with relevant current references. Any changes to the reference list should be mentioned in the rebuttal letter that accompanies your revised manuscript. If you need to cite a retracted article, indicate the article’s retracted status in the References list and also include a citation and full reference for the retraction notice.

**Additional Editor Comments:**

**Please address final minor comments.**

[Note: HTML markup is below. Please do not edit.]

Reviewers’ comments:

Reviewer’s Responses to Questions

**Comments to the Author**

Reviewer #1: All comments have been addressed

Reviewer #2: All comments have been addressed

Reviewer #3: All comments have been addressed

**********

2. Is the manuscript technically sound, and do the data support the conclusions?

Reviewer #1: Yes

Reviewer #2: Yes

Reviewer #3: Yes

**********

3. Has the statistical analysis been performed appropriately and rigorously?

Reviewer #1: Yes

Reviewer #2: Yes

Reviewer #3: Yes

**********

4. Have the authors made all data underlying the findings in their manuscript fully available?

The PLOS Data policy

Reviewer #1: No

Reviewer #2: Yes

Reviewer #3: Yes

**********

5. Is the manuscript presented in an intelligible fashion and written in standard English?

Reviewer #1: Yes

Reviewer #2: Yes

Reviewer #3: Yes

**********

Reviewer #1: Authors have worked well to address the comments

Authors have worked well to address the comments

Suggest acceptance

Reviewer #2: Authors exhaustively addressed prior concerns raised by the Reviewers.

They acknowledged some limitations, that surely limits the generalizability of the findings.

Reviewer #3: 1 - The comments made addressing prior reviewer comments I believe are appropriate. I do not have much else to add from the edits that were already made from the other reviewers. Thank you for making those.

2 - Overall, this is a very interesting study. I personally believe that as technology broadens and more reliable/accurate non-invasive tools (such as salivary biomarker testing) are available to patients, that we clinically would be able to offer better care. Anecdotally in my practice, many patients fear serum measurements. Patient’s also have multiple specialists/providers ordering different labs requiring patients to have venipunctures frequently which contributes to the burden of simply being a patient. I applaud your team for looking into alternatives and the theory of salivary testing I hope leads to breakthroughs in the future. One comment I have is regarding the conclusion that saliva should be considered a "complementary matrix" for cardiovascular biomarker assessment. The data do not clearly support this framing. All three biomarkers showed weak, non-significant correlations between salivary and serum concentrations at all time points, and Bland-Altman analyses demonstrated clinically unacceptable agreement for NT-proBNP and troponin. While hs-CRP showed a mean bias closer to zero, the limits of agreement remained wide and inter-individual variability was substantial. Given these findings, it is unclear in what clinical scenario salivary measurements would meaningfully complement serum-based assessment. If you believe that salivary testing can complement serum testing, you should either provide a specific, clinically grounded example of how salivary biomarkers could add value alongside serum measurements, or adjust the language to more accurately reflect the findings - characterizing saliva as a matrix warranting further investigation rather than a current complementary tool.

3 - inclusion of patients with Stage II or III periodontitis as an eligibility criterion deserves mention as a potential limitation on external validity, as periodontal disease may influence salivary composition and biomarker concentrations. While this was driven by the design of the parent study, it restricts the applicability of findings to the broader heart failure population. This potentially opens up the door for having the study repeated in a population without periodontal disease.

4 - One methodological gap worth noting is the absence of pre-specified, clinically acceptable limits of agreement prior to conducting the Bland-Altman analysis. In a measurement agreement study, defining acceptable thresholds prior to strengthens the interpretability of results. Without this, the determination that agreement is "clinically unacceptable" for NT-proBNP and troponin - while intuitively supported by the data - rests on post-hoc visual and qualitative judgment rather than a prospectively defined criterion. The missing piece is a pre-specified clinical threshold that says "we will only accept saliva as a comparable matrix if the differences between methods fall within X." That threshold needs to come from clinical judgment before looking at the data, not from the statistical output itself.

**********

what does this mean?). If published, this will include your full peer review and any attached files.

If you choose “no”, your identity will remain anonymous but your review may still be made public.

**Do you want your identity to be public for this peer review?**  For information about this choice, including consent withdrawal, please see our Privacy Policy

Reviewer #1: **Yes:** Luca Fazzini

Reviewer #2: No

Reviewer #3: No

**********

[NOTE: If reviewer comments were submitted as an attachment file, they will be attached to this email and accessible via the submission site. Please log into your account, locate the manuscript record, and check for the action link "View Attachments". If this link does not appear, there are no attachment files.]

To ensure your figures meet our technical requirements, please review our figure guidelines: https://journals.plos.org/plosone/s/figures

You may also use PLOS’s free figure tool, NAAS, to help you prepare publication quality figures: https://journals.plos.org/plosone/s/figures#loc-tools-for-figure-preparation.

NAAS will assess whether your figures meet our technical requirements by comparing each figure against our figure specifications.

# Author comment: Author response to Decision Letter 2

**DOI:** 10.1371/journal.pone.0357206.r004

22 Jun 2026

Uploaded file named as answering letter. R2.

Attachment Submitted filename: 16-6-2026 answering letter R2 .docx

# Editor report: Decision Letter 2

**DOI:** 10.1371/journal.pone.0357206.r005

**Authors:** Michele Golino

19 Jul 2026

Dear Dr. cortelli,

==========================

==========================

Please submit your revised manuscript by Sep 02 2026 11:59PM. If you will need more time than this to complete your revisions, please reply to this message or contact the journal office at plosone@plos.org. When you’re ready to submit your revision, log on to https://www.editorialmanager.com/pone/ and select the ‘Submissions Needing Revision’ folder to locate your manuscript file.

Please include the following items when submitting your revised manuscript:

- A letter that responds to each point raised by the academic editor and reviewer(s). You should upload this letter as a separate file labeled ‘Response to Reviewers’.
- A marked-up copy of your manuscript that highlights changes made to the original version. You should upload this as a separate file labeled ‘Revised Manuscript with Track Changes’.
- An unmarked version of your revised paper without tracked changes. You should upload this as a separate file labeled ‘Manuscript’.

If you would like to make changes to your financial disclosure, please include your updated statement in your cover letter. Guidelines for resubmitting your figure files are available below the reviewer comments at the end of this letter.

If applicable, we recommend that you deposit your laboratory protocols in protocols.io to enhance the reproducibility of your results. Protocols.io assigns your protocol its own identifier (DOI) so that it can be cited independently in the future. For instructions see: https://journals.plos.org/plosone/s/submission-guidelines#loc-laboratory-protocols. Additionally, PLOS ONE offers an option for publishing peer-reviewed Lab Protocol articles, which describe protocols hosted on protocols.io. Read more information on sharing protocols at https://plos.org/protocols?utm_medium=editorial-email&utm_source=authorletters&utm_campaign=protocols.

As the corresponding author, your ORCID iD is verified in the submission system and will appear in the published article. PLOS supports the use of ORCID, and we encourage all coauthors to register for an ORCID iD and use it as well. Please encourage your coauthors to verify their ORCID iD within the submission system before final acceptance, as unverified ORCID iDs will not appear in the published article. *Only*  the individual author can complete the verification step; PLOS staff *cannot*  verify ORCID iDs on behalf of authors.

We look forward to receiving your revised manuscript.

Kind regards,

Michele Golino, M.D., Ph.D.

Academic Editor

PLOS One

Journal Requirements:

If the reviewer comments include a recommendation to cite specific previously published works, please review and evaluate these publications to determine whether they are relevant and should be cited. There is no requirement to cite these works unless the editor has indicated otherwise.

Please review your reference list to ensure that it is complete and correct. If you have cited papers that have been retracted, please include the rationale for doing so in the manuscript text, or remove these references and replace them with relevant current references. Any changes to the reference list should be mentioned in the rebuttal letter that accompanies your revised manuscript. If you need to cite a retracted article, indicate the article’s retracted status in the References list and also include a citation and full reference for the retraction notice.

Additional Editor Comments:

Dear Authors,

Thank you for your careful revision of the manuscript.

You have addressed the previous reviewer comments thoughtfully, and the overall quality of the paper has improved substantially.

The remaining points are relatively minor and mainly relate to clarifying the interpretation of your findings, moderating some of the conclusions to better align with the presented data, and explicitly acknowledging methodological limitations, including the restricted external validity of the study population and the absence of pre-specified clinically acceptable limits of agreement.

I believe addressing these points will further strengthen the manuscript.

[Note: HTML markup is below. Please do not edit.]

[NOTE: If reviewer comments were submitted as an attachment file, they will be attached to this email and accessible via the submission site. Please log into your account, locate the manuscript record, and check for the action link "View Attachments". If this link does not appear, there are no attachment files.]

To ensure your figures meet our technical requirements, please review our figure guidelines: https://journals.plos.org/plosone/s/figures

You may also use PLOS’s free figure tool, NAAS, to help you prepare publication quality figures: https://journals.plos.org/plosone/s/figures#loc-tools-for-figure-preparation.

NAAS will assess whether your figures meet our technical requirements by comparing each figure against our figure specifications.

# Author comment: Author response to Decision Letter 3

**DOI:** 10.1371/journal.pone.0357206.r006

11 Aug 2026

Attached as a separet file.

Attachment Submitted filename: 1662026 answering letter R3.docx

# Editor report: Decision Letter 3

**DOI:** 10.1371/journal.pone.0357206.r007

**Authors:** Michele Golino

14 Aug 2026

Salivary and Serum Levels of NT-proBNP, Troponin, and High-Sensitivity C-Reactive Protein in Patients with Heart Failure

PONE-D-26-06014R3

Dear Dr. cortelli,

We’re pleased to inform you that your manuscript has been judged scientifically suitable for publication and will be formally accepted for publication once it meets all outstanding technical requirements.

Within one week, you’ll receive an e-mail detailing the required amendments. When these have been addressed, you’ll receive a formal acceptance letter and your manuscript will be scheduled for publication.

An invoice will be generated when your article is formally accepted. Please note, if your institution has a publishing partnership with PLOS and your article meets the relevant criteria, all or part of your publication costs will be covered. Please make sure your user information is up-to-date by logging into Editorial Manager at Editorial Manager® and clicking the ‘Update My Information’ link at the top of the page. For questions related to billing, please contact billing support.

If your institution or institutions have a press office, please notify them about your upcoming paper to help maximize its impact. If they’ll be preparing press materials, please inform our press team as soon as possible -- no later than 48 hours after receiving the formal acceptance. Your manuscript will remain under strict press embargo until 2 pm Eastern Time on the date of publication. For more information, please contact onepress@plos.org.

Kind regards,

Michele Golino, M.D., Ph.D.

Academic Editor

PLOS One

Additional Editor Comments (optional):

Dear Authors,

thank you for resubmitting the last version of your manuscript and for having addressed the last minor comments.

The manuscript has substantially improved.

Reviewers’ comments:

# Editor report: Acceptance letter

**DOI:** 10.1371/journal.pone.0357206.r008

**Authors:** Michele Golino

PONE-D-26-06014R3

PLOS One

Dear Dr. Cortelli,

I’m pleased to inform you that your manuscript has been deemed suitable for publication in PLOS One. Congratulations! Your manuscript is now being handed over to our production team.

At this stage, our production department will prepare your paper for publication. This includes ensuring the following:

* All references, tables, and figures are properly cited

* All relevant supporting information is included in the manuscript submission,

* There are no issues that prevent the paper from being properly typeset

You will receive further instructions from the production team, including instructions on how to review your proof when it is ready. Please keep in mind that we are working through a large volume of accepted articles, so please give us a few days to review your paper and let you know the next and final steps.

Lastly, if your institution or institutions have a press office, please let them know about your upcoming paper now to help maximize its impact. If they’ll be preparing press materials, please inform our press team within the next 48 hours. Your manuscript will remain under strict press embargo until 2 pm Eastern Time on the date of publication. For more information, please contact onepress@plos.org.

You will receive an invoice from PLOS for your publication fee after your manuscript has reached the completed accept phase. If you receive an email requesting payment before acceptance or for any other service, this may be a phishing scheme. Learn how to identify phishing emails and protect your accounts at https://explore.plos.org/phishing.

If we can help with anything else, please email us at customercare@plos.org.

Thank you for submitting your work to PLOS One and supporting open access.

Kind regards,

PLOS One Editorial Office Staff

on behalf of

Dr. Michele Golino

Academic Editor

PLOS One
